# Alteration of *SF3B1* and *SRSF2* Genes in Myelodysplastic Syndromes Patients in Upper Northern Thailand

**DOI:** 10.31557/APJCP.2019.20.4.1215

**Published:** 2019

**Authors:** Phuttirak Yimpak, Adisak Tantiworawit, Thanawat Rattanathammethee, Sirinda Angsuchawan, Sikrai Laowatthanapong, Witoon Tasuya, Kanokkan Bumroongkit

**Affiliations:** 1 *Department of Anatomy, *; 2 *Division of Hematology, Department of Internal Medicine , Faculty of Medicine, Chiang Mai University, Chiang Mai, Thailand.*

**Keywords:** Myelodysplastic syndrome, SF3B1 gene mutation, SRSF2 gene mutation, splicing machinery gene

## Abstract

**Background:**

The frequency and pattern of mutation in *SF3B1* and *SRSF2* RNA splicing machinery genes were found to vary among myelodysplastic syndrome (MDS) patients in different populations. There have been less reports of incidence of these gene mutations in Thailand especially in upper northern Thailand. This study therefore had aims to investigate the frequency and pattern of mutation in mutational hotspot of *SF3B1* and *SRSF2* genes among MDS patients in upper northern Thailand and to investigate the clinical features associated with the mutations.

**Methods:**

Fifty-five MDS patients who underwent treatment at Maharaj Nakorn Chiang Mai Hospital participated in this study. The detection of *SF3B1* and *SRSF2* hotspot mutations was carried out using polymerase chain reaction followed by Sanger sequencing. In addition, clinical features of individual patients with these gene mutations were also investigated.

**Results:**

*SF3B1* mutations (*SF3B1*^mut^) were found in 9 patients (16.4%) including E622D (1/9), R625C (1/9), H662Q (1/9), K700E (5/9), and Q699H co-mutation with K700E (1/9). *SRSF2* mutations (*SRSF2*^mut^) were found in 4 patients (7.3%) which included P95H (3/4) and P95L (1/4). The *SF3B1*^mut^ was associated with lower hemoglobin levels (*p* = 0.023) and higher platelet counts (*p* = 0.047) when compared with MDS patients without *SF3B1*^mut^, while *SRSF2*^mut^ tended to occur in patients with a higher percentage of bone marrow blasts (*p* = 0.074).

**Conclusion:**

The findings confirmed the difference in frequency of *SF3B1* and *SRSF2* mutations among different populations. Specifically, we found a co-mutation of Q699H and K700E that has not been previously reported in MDS patients in the COSMIC database. It was also found that *SF3B1*^mut^ was strongly associated with low hemoglobin level, and high platelet counts whereas *SRSF2*^mut^ was mostly clustered in MDS with excess blasts subsequently increasing the probability of progression to acute myeloid leukemia.

## Introduction

Myelodysplastic syndromes (MDS) has been defined as a group of clonal bone marrow (BM) disorders characterized by ineffective hematopoiesis which contributes to morphologic dysplasia in hematopoietic cells and peripheral blood cytopenia(s) (Arber et al., 2016). Currently, the mutations in RNA splicing machinery genes have been reported as being concretely proportional in MDS patients. The RNA splicing is a key to the regulation of gene expression. Intact and accurate RNA splicing is essential for the accuracy of final protein products. Any alterations in these pathways contribute to the dysfunction of the final protein products and subsequently are the cause of disease. *SF3B1*, one of the RNA splicing machinery genes, is located on chromosome 2q33.1. This gene encodes the subunit 1 of the splicing factor 3b complex which is an essential component of the U2snRNP complex and is important for the recognition of the 3’ splice site between intron and exon in normal RNA splicing (Padgett, 2012; Cazzola et al., 2013). *SF3B1*^mut^ is frequently found in MDS patients and is associated with the presence of ring sideroblasts (RS) which are erythroid precursors showing iron deposition in the mitochondria cover around the nuclear circumference (Papaemmanuil et al., 2011; Cazzola et al., 2013). Currently, in the World Health Organization (WHO) 2016 guidelines, *SF3B1*^mut^ is used as a biomarker for the classification of MDS (Arber et al., 2016). Several studies have reported that MDS with *SF3B1*^mut^ are associated with a favorable prognosis (Malcovati et al., 2011; Papaemmanuil et al., 2011; Cui et al., 2012). In addition to *SF3B1*, *SRSF2*^mut^ are commonly detected in MDS patients. *SRSF2* is located on chromosome 17q25.1 and encodes for the serine/arginine rich splicing factor 2 (SRSF2) which is associated with the regulation of constitutive and alternative pre-mRNA splicing (Long and Caceres, 2009; Wu et al., 2012). *SRSF2*^mut^ has an unfavorable impact on MDS patients and its presence predicts shorter overall survival when compared with MDS patients without *SRSF2*^mut^ (Thol et al., 2012). As the regulation of RNA splicing is essential for normal functioning of the cell, the alteration in *SF3B1* and *SRSF2* splicing machinery genes may certainly be involved in the pathogenesis of MDS. There have been few studies regarding *SF3B1* and *SRSF2* gene mutations and clinical features of MDS patients in Thailand especially in upper northern Thailand. Therefore, this study aimed to investigate the frequency and patterns of the mutations along with the clinical features including RS in *SF3B1* and *SRSF2* gene mutations among MDS patients in upper northern Thailand.

## Materials and Methods

*Patients*

From 2017 – 2018, a total of 55 BM samples and 37 dried BM smear slides of MDS patients with ≥ 18 years old from the Division of Hematology, Department of Internal Medicine, Maharaj Nakorn Chiang Mai Hospital (Chiang Mai, Thailand) were recruited onto the study. All of patients were diagnosed MDS confirmed by a hematologist according to WHO 2016 classification criteria. Clinical features and hematological data were collected from electronic medical records. This study was approved by the Ethics and Research Committee of the Faculty of Medicine, Chiang Mai University [Certificate No. 327 /2017 Study code ANA-2560-04844].

*Prussian blue staining for detecting ring sideroblasts (RS)*

The protocol was modified from Jouihan (2012) (Jouihan, 2012). Dried BM smear slides were immersed in a freshly mixed solution of 2% potassium ferrocyanide and 2% HCl in equal proportions and counterstained with nuclear fast red. One hundred erythroid cells were counted for each specimen (Mufti et al., 2008). RS was defined by the presence of at least five siderotic granules extending over at least one third of the nucleus circumference in a stained BM smeared slide (Lee et al., 2008). The presence of ≥15% RS from 100 erythroid cells was defined as positive for RS.

*Detection of SF3B1 exon 14, 15 and SRSF2 exon 1 mutation*

Genomics DNA was extracted using inorganic salting out method modified from Seielstad et al., (1999)(Seielstad et al., 1999) and the QIAamp^®^ DNA Mini Kit (QIAGEN, Hilden, Germany) following the manufacturer’s instructions. The genomic DNA was amplified by using polymerase chain reaction (PCR) targeting the mutational hotspot of *SF3B1* (exon 14 and 15) and *SRSF2* (exon 1). The sequences of each primer are shown in Table 1. The PCR process was as follows: initial denaturation step at 95°C for 1 minute, followed by 35 cycles of denaturation at 95°C for 15 seconds, annealing at 60°C for 15 seconds, and extension at 72°C for 10 seconds on a thermocycler (Eppendorf Mastercycler, USA). PCR products were purified using NucleoSpin^® ^Gel and PCR Clean-up (Macherey-Nagel, Germany) and sequenced bidirectionally using the BigDye^® ^Terminator Version 3.1 Cycle Sequencing Kit (Applied Biosystems, Foster City, California, USA). Sequencing reactions were purified using the Ethanol/EDTA/Sodium acetate precipitation method and run on the ABI Prism 3130^® ^DNA Analyzer (Applied Biosystems, Foster City, California, USA). The electropherograms were analyzed using the Seqscape program V2.5 (Applied Biosystems, Foster City, California, USA) to detect the mutations by comparing the resultant products with reference DNA sequences from Genbank (NG_032903.2 for *SF3B1* gene and NG_032905.1 for *SRSF2* gene). In all cases of mutational detection, PCR and sequencing were repeated to confirm the results.

*Statistical analysis*

The frequencies of gene mutation were counted and percentages calculated. The Mann-Whitney U test or Student’s *t*-test was used for the comparison of numerical variables (such as age, BM blast, white blood cell count (WBC), absolute neutrophil count (ANC), platelet count, and hemoglobin between groups. Fisher’s exact test or the Chi-square test was performed to analyze the significance of the association between gene mutations and categorical variable parameters, such as sex, WHO classification, RS, cytogenetic categories and IPSS-R categories. A *p*-value less than 0.05 was considered to be statistically significant. 

## Results

*Clinical data and laboratory feature of all 55 MDS patients*

**Figure 1:**
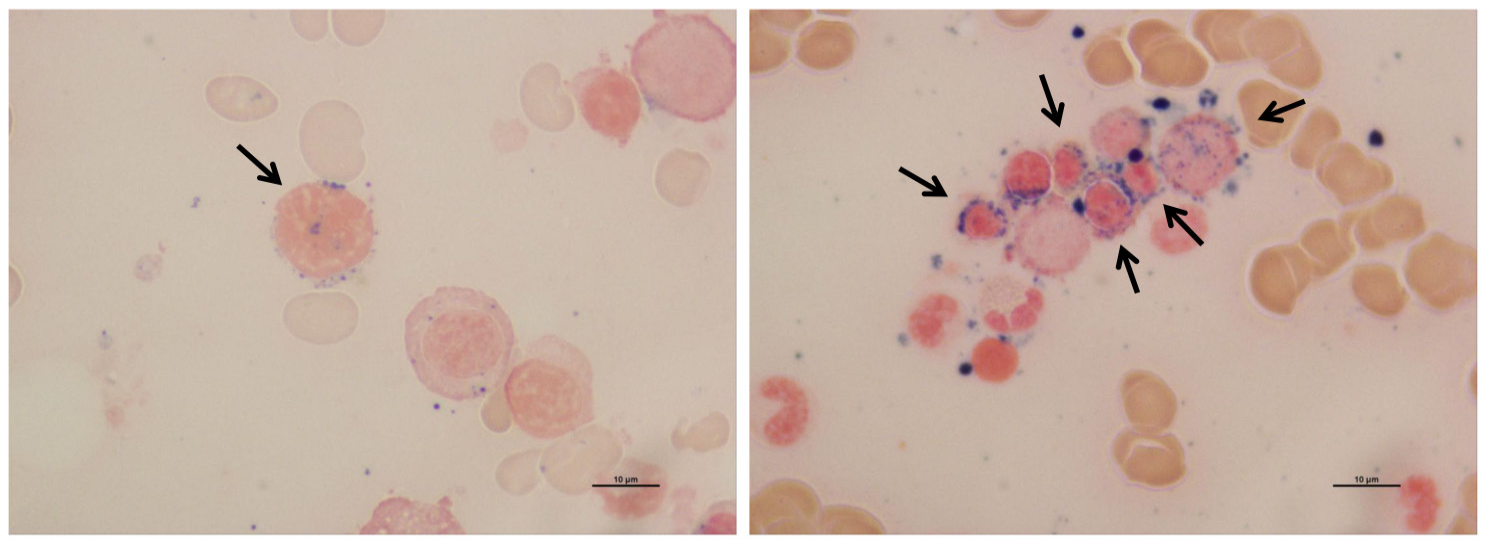
RS in Bone Marrow Smears with Prussian Blue Staining Technique: ×1000; Black Arrow, RS

The characteristics of 55 upper northern Thai MDS patients are shown in Table 2. Out of the 55 patients, 37 patients had BM smeared slides for RS detection. RS was positive in 4 patients (10.8%). The morphology of RS is shown in Figure 1. 

**Table 1:** The Primers for PCR Amplification and Sequencing of *SF3B1* and *SRSF2* Genes

*SF3B1*				
For PCR	Exon 14-15	Forward	TAGAGTGGAAGGCCGAGAGA	(Kang et al., 2015)
		Reverse	TTCAAGAAAGCAGCCAAACC	(Kang et al., 2015)
For Sequencing	Exon 14	Forward	TAGAGTGGAAGGCCGAGAGA	(Kang et al., 2015)
		Reverse	CAACTTACCATGTTCAATGATTTC	(Malcovati., 2011)
	Exon 15	Forward	GTTGATATATTGAGAGAATC	(Kang et al., 2015)
		Reverse	TTCAAGAAAGCAGCCAAACC	(Kang et al., 2015)
*SRSF2*				
For PCR and sequencing	Exon 1	Forward	GTGGACAACCTGACCTACCG	(Kang et al., 2015)
		Reverse	CCTCAGCCCCGTTTACCT	(Kang et al., 2015)

**Figure 2:**
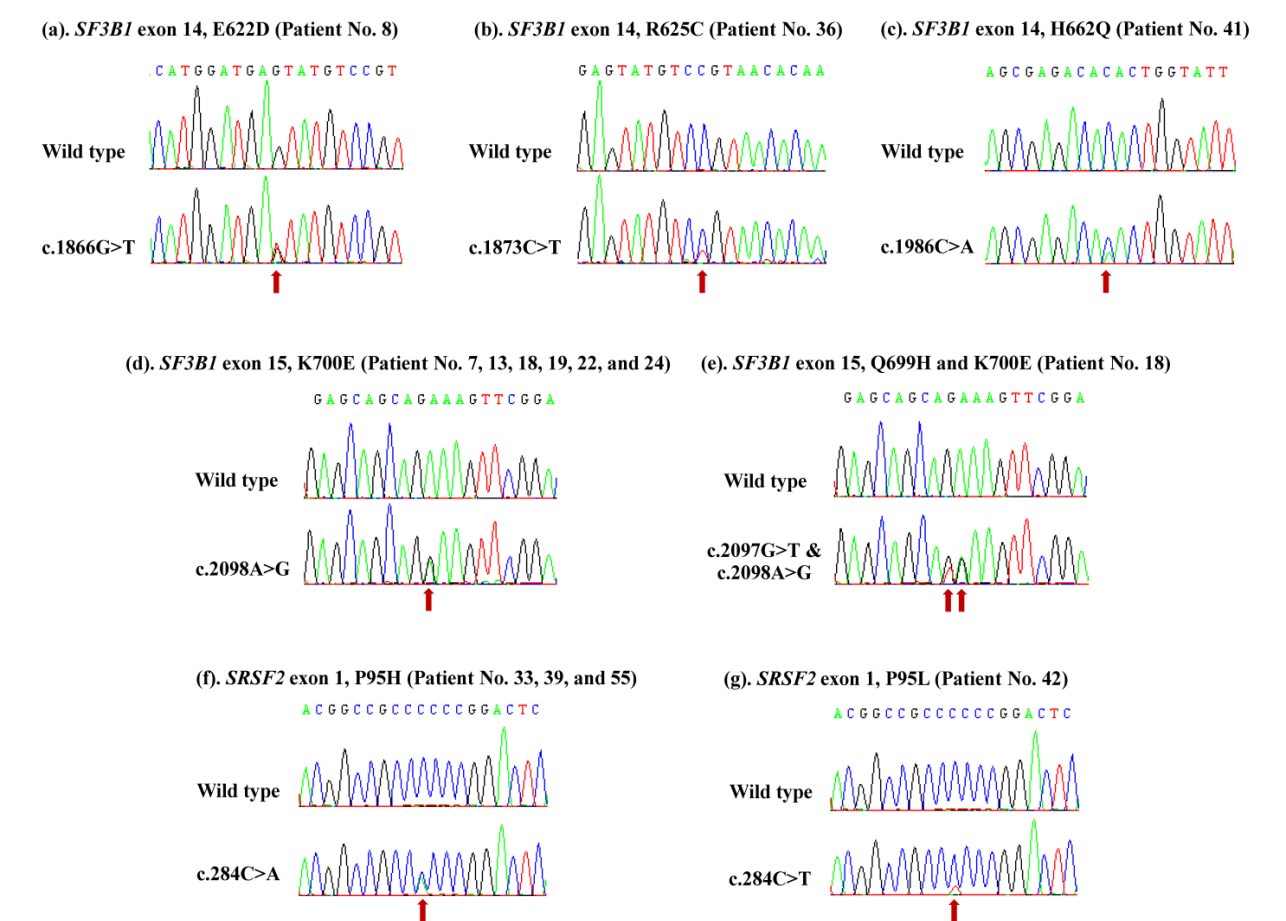
Electropherograms by Sanger sequencing show mutations in splicing machinery genes: (a), (b), and (c), *SF3B1*^mut^ in exon 14; (d) and (e), *SF3B1*^mut^ in exon 15; (f) and (g), *SRSF2*^mut^ in exon 1; Red arrow, point of the location of base substitution

**Figure 3:**
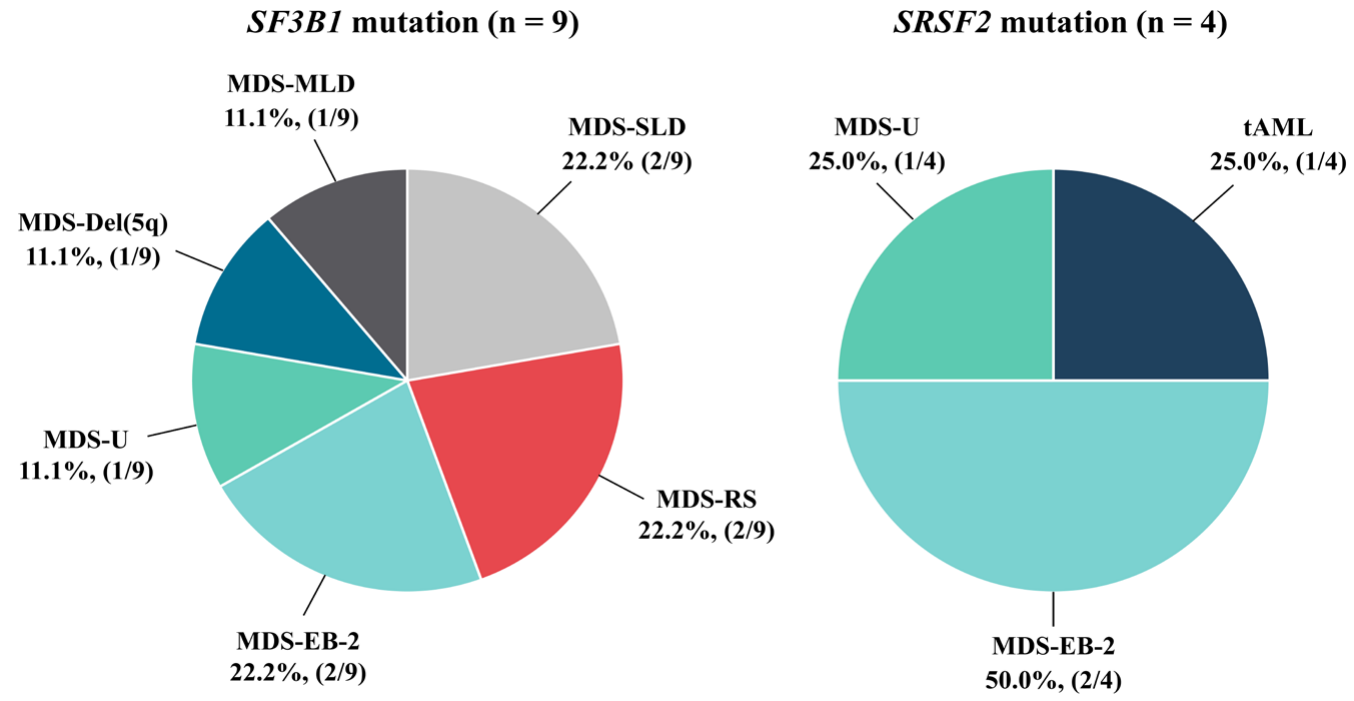
World Health Organization Classification of MDS Patients with *SF3B1* and *SRSF2 *Mutation

**Figure 4:**
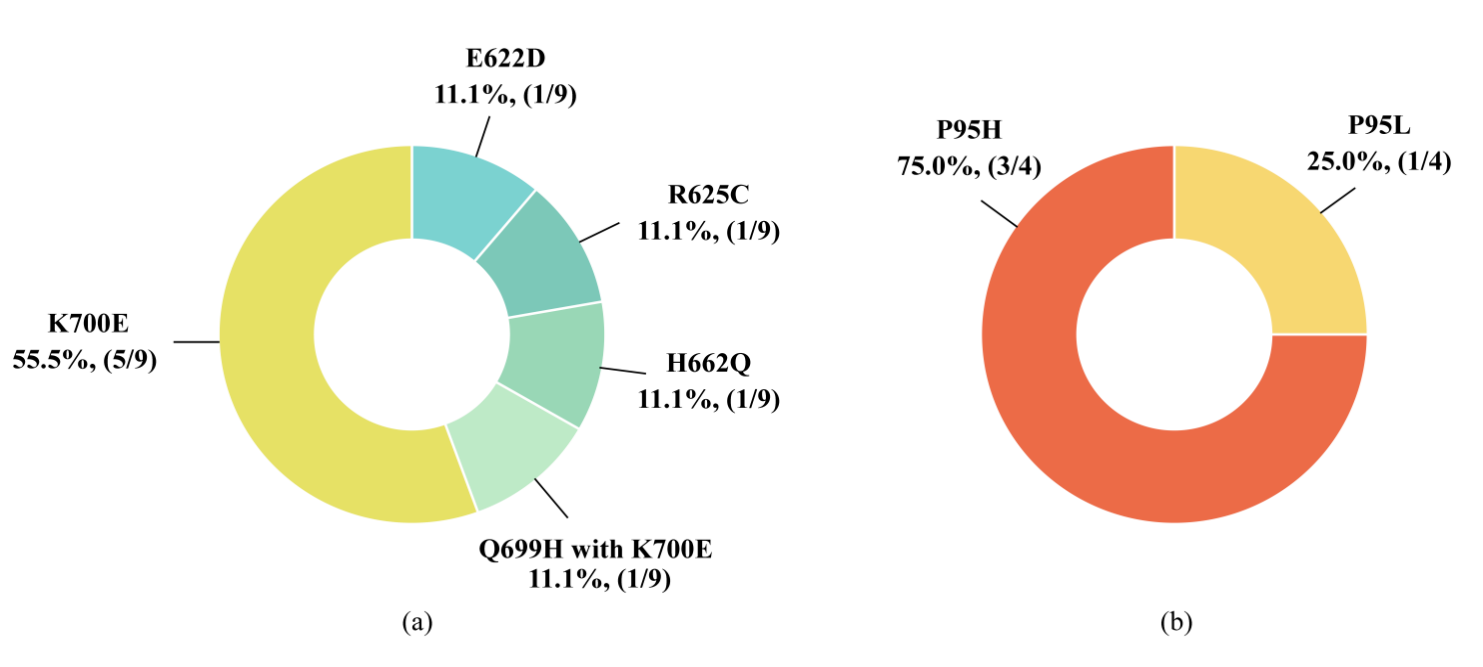
Pattern and Frequency of *SF3B1* and *SRSF2 *Mutations in MDS Patients (a) Pattern and frequency of *SF3B1*^mut^*;* (b) Pattern and frequency of *SRSF2*^mut^

**Table 2:** Clinical Characteristics of MDS Patients According to the Alteration of *SF3B*1 and *SRSF2* Status

Characteristics	All patients (n = 55)	*SF3B1* wild type (n = 46, 83.6%)	*SF3B1* mutation (n = 9, 16.4%)	*P*	*SRSF2* wild type (n = 51, 92.7%)	*SRSF2* mutation (n = 4, 7.3%)	*P*
Median Age (years), (range)	65 (31-93)	65 (31-93)	62 (42-76)	0.358	65 (31-91)	67 (56-93)	0.484
Sex				0.281			0.613
Male, n (%)	29 (52.7)	26 (56.5)	3 (33.3)		26 (51.0)	3 (75.0)	
Female, n (%)	26 (47.3)	20 (43.5)	6 (66.7)		25 (49.0)	1 (25.0)	
WHO classification, n (%)				0.042*			0.372
MDS-SLD	11 (20.0)	9 (19.6)	2 (22.2)		11 (21.6)	-	
MDS-MLD	14 (25.5)	13 (28.3)	1 (11.1)		14 (27.5)	-	
MDS-RS	2 (3.6)	-	2 (22.2)		2 (3.9)	-	
MDS with isolate del(5q)	1 (1.8)	-	1 (11.1)		1 (2.0)	-	
MDS-EB-1	2 (3.6)	2 (4.3)	-		2 (3.9)	-	
MDS-EB-2	10 (18.2)	8 (17.4)	2 (22.2)		8 (15.7)	2 (50.0)	
MDS-U	10 (18.2)	9 (19.6)	1 (11.1)		9 (17.6)	1 (25.0)	
Transformation to AML	5 (9.1)	5 (10.8)	-		4 (7.8)	1 (25.0)	
Blood counts, median (range)^a^ (n=50)							
BM blasts (%)	1.0 (1.0 - 19.0)	1.0 (0.0 – 19.0)	1.0 (0.0 -12.0)	0.467	1.0 (0.0 - 19.0)	13.0 (1.0 – 19.0)	0.074
WBC (×10^9^/L)	4.4 (0.7 – 47.7)	4.4 (0.7 – 47.7)	5.3 (2.3 – 13.0)	0.92	4.4 (0.7 – 18.2)	5.5 (4.0 – 47.7)	0.213
ANC (×10^9^/L)	4.2 (0.9 – 9.1)	4.3 (0.9 – 9.1)	2.7 (2.2 – 5.0)	0.116	4.2 (0.9 – 9.1)	4.0 (4.0 – 5.7)	0.61
Hemoglobin (g/dL)	8.5 (4.2 – 13.4)	8.7 (6.2 – 13.4)	6.7 (4.2 – 10.1)	0.023*	8.5 (4.2 – 13.4)	8.7 (6.2 – 8.9)	0.591
Platelets (×10^9^/L)	66.0 (5.0 – 485.0)	58.0 (5.0-485.0)	129.0 (36.0-290.0)	0.047*	62.0 (5.0 – 485.0)	139.0 (77.0 – 307.0)	0.111
Cytogenetic categories, n (%)				1			0.137
Very good	3 (5.5)	3 (6.5)	-		3 (5.9)	-	
Good	39 (70.9)	32 (69.6)	7 (77.8)		37 (72.5)	2 (50.0)	
Intermediate	6 (10.9)	5 (10.9)	1 (11.1)		5 (9.8)	1 (25.0)	
Poor	2 (3.6)	2 (4.3)	-		2 (3.9)	-	
Very poor	4 (7.3)	3 (6.5)	1 (11.1)		4 (7.8)	-	
N/A	1 (1.8)	1 (2.2)	-		-	1 (25.0)	
IPSS-R categories, n (%)				0.978			0.117
Very low	7 (12.7)	6 (13.0)	1 (11.1)		7 (13.7)	-	
Low	25 (45.5)	20 (43.5)	5 (55.6)		24 (47.1)	1 (25.0)	
Intermediate	5 (9.1)	4 (8.7)	1 (11.1)		5 (9.8)	-	
High	7 (12.7)	6 (13.0)	1 (11.1)		6 (11.8)	1 (25.0)	
Very high	10 (18.2)	9 (19.6)	1 (11.1)		9 (17.6)	1 (25.0)	
N/A	1 (1.8)	1 (2.2)	-		-	1 (25.0)	
RS, (n=37)				0.05			0.207
Present (n)	4 (10.8)	2 (6.1)	2 (50.0)		3 (8.6)	1 (50.0)	
Not present (n)	33 (89.2)	31 (93.9)	2 (50.0)		32 (91.4)	1 (50.0)	

^a^, Patient with transformation to AML were excluded from analysis (n=50); *, *p*-value less than 0.05 was considered statistically significant; MDS-SLD, Myelodysplastic syndrome with single lineage dysplasia; MDS-MLD, Myelodysplastic syndrome with multilineage dysplasia; MDS-RS, Myelodysplastic syndrome with ring sideroblasts; MDS-EB-1, Myelodysplastic syndrome with excess blast – 1; MDS-EB-2, Myelodysplastic syndrome with excess blast – 2; MDS-U, Myelodysplastic syndrome unclassifiable; Transformation to AML, transformation to acute myeloid leukemia; BM blasts, bone marrow blasts; WBC, white blood cells; RS, ring sideroblasts; IPSS-R, Revised International Prognostic Scoring System; N/A, not available.

**Table 3:** Alteration of Mutations in *SF3B1* and *SRSF2* Genes of MDS Patients and the Amino Acid Changes

Gene	Exon	Mutation	Amino acid change	Pattern of mutation	Frequency (%)
*SF3B1*	14	c.1866G>T	p.E622D	Missense substitution	1/9 (11.1)
		c.1873C>T	p.R625C	Missense substitution	1/9 (11.1)
		c.1986C>A	p.H662Q	Missense substitution	1/9 (11.1)
	15	c.2098A>G	p.K700E	Missense substitution	5/9 (55.5)
		c.2097G>T & c.2098A>G	p.Q699H & K700E	Missense substitution	1/9 (11.1)
*SRSF2*	1	c.284C>A	p.P95H	Missense substitution	3/4 (75.0)
		c.284C>T	p.P95L	Missense substitution	1/4 (25.0)

The clinical data of patients with relation to *SF3B1*^mut^ and *SRSF2*^mut ^status are shown in Table 2. The *SF3B1*^mut^ were found in 9 patients (16.4%), one of the 9 patients having 2 affected codons. The *SRSF2*^mut^ were found in 4 patients (7.3%). There was a statistically significant difference in the World Health Organization classification between patients with *SF3B1*^mut ^and without* SF3B1*^mut^ (*p* = 0.042). On analysis of the clinical data (blood count), patients with transformation to AML were excluded. Patients with *SF3B1*^mut^ had lower hemoglobin levels (*p* = 0.023) and higher platelet counts (*p* = 0.047) than those patients without *SF3B1*^mut^. There was no obvious correlation in age, sex, bone marrow blasts, white blood cell counts, absolute neutrophil counts, cytogenetic category, and IPSS-R category between patients with *SF3B1*^mut^ and without* SF3B1*^mut^*.* In the patients with *SF3B1*^mut^ there seemed to be an association with ring sideroblasts, however, there was no significant difference (*p* = 0.050). As regards *SRSF2*^mut^, there was no significant difference in age, sex, World Health Organization classification, bone marrow blasts, white blood cell count, absolute neutrophil count, hemoglobin, platelet counts, ring sideroblasts, cytogenetic category, and IPSS-R category between patients with and without *SRSF2*^mut ^(Table 2).

*Frequency and pattern of SF3B1* ^mut^ *and SRSF2* ^mut^ *in MDS patients*

Mutations in the *SF3B1* and *SRSF2* splicing machinery genes were detected in 13 out of 55 MDS patients (23.6%). We found *SF3B1*^mut^ in 9 patients (16.4%), one of the 9 patients had 2 affected codons. *SRSF2*^mut^ were found in 4 patients (7.3%) (Table 2). These MDS patients displayed either an *SF3B1* or *SRSF2 *gene mutation. None of patients carried both gene mutations. All the mutations of these genes were heterozygous missense mutations (Table 3 and Figure 2). Patients with *SF3B1*^mut^ were 2 MDS-SLD (22.2 %), 1 MDS-MLD (11.1 %), 2 MDS-RS (22.2 %), 1 MDS with isolated del(5q) (11.1 %), 2 MDS-EB-2 (22.2 %), and 1 MDS-U (11.1%) (Table 2 and Figure 3). We found 10 affected codons in 9 patients including E622, R625, H662, Q699, and K700 (Table 3, Figure 2 and 4). The K700 mutation was the most common *SF3B1*^mut^ observed in this study which was positive in 6 patients. Additionally, one out of these patients displayed a co-mutation with Q699. Regarding *SRSF2*^mut^, the mutations were found in MDS-EB-2, MDS-U subtype and transformation to AML (tAML) for 2 (50.0%), 1 (25.0%) and 1 (25.0%) patients respectively (Table 2 and Figure 3). All of these showed heterozygous missense mutations in codon P95 (Figure 2 and 4). 

## Discussion

The mutations in RNA splicing machinery genes have been reported in a concrete proportion of adult MDS patients with varying frequencies between the different populations (Yoshida et al., 2011; Damm et al., 2012; Thol et al., 2012; Kang et al., 2015). The incidence of these gene mutations have been less frequently reported in Thailand especially in the upper northern population. We identified the alteration of *SF3B1* and *SRSF2* genes at the mutational hotspot exon by the PCR technique followed by Sanger sequencing. The results showed the frequency of these gene mutations were different to those in other populations and the mutations were associated with some clinical data.

*SF3B1* ^mut^ at exon 14 – 15 was detected in 9 out of 55 patients (16.4%). When considering the frequency of these exons mutation in other populations, *SF3B1*^mut ^were found in 6.6% (6/91) of Brazilian patients (Donaires et al., 2016) which is lower than this study. In Asian populations the frequency of *SF3B1*^mut^ was different from this report, including a 52.9% (55/104) incidence in Chinese patients (Cui et al., 2012); 10.0% (48/479) in Taiwanese patients (Lin et al., 2014a), and 7.0% (9/129) in Korean patients (Kang et al., 2015). However, the frequency of these gene mutations in our study was similar to that reported in a study by Rujirachaivej (2018) which cited 13.9% (10/72) in Thai MDS patients who were undergoing treatment at Ramathibodi Hospital located in central Thailand (Rujirachaivej et al., 2018). The various in frequencies and pattern of *SF3B1* gene mutations among different population could depend on many factors such as diversity of genetic background, heterogeneity of disease, environment, individual lifestyle or the number of samples. Individual laboratory methods may also contribute to these differences. The people in upper northern Thailand experience different environment factors and come from a different genetic pool to those in other parts of Thailand. The information regarding *SF3B1*^mut^ in MDS in the upper northern Thai population might be of further use for diagnosis and prognosis in this patient group.

This study detected five different *SF3B1* missense mutations in 9 patients (Table 3). K700E is the most frequent mutation pattern of *SF3B1* in this study, a similar finding to prior studies (Papaemmanuil et al., 2011; Cui et al., 2012; Patnaik et al., 2012; Seo et al., 2014; Donaires et al., 2016). Codon K700 is located on exon 15 of the *SF3B1* gene, and the K700E missense mutation leads to a lysine to glutamic acid substitution. The other affected pattern on exon 15 is Q699H, resulting in a glutamine to histidine substitution and this affected codon co-occurring with K700E. A co-mutation of Q699H and K700E that has not been previously reported in MDS patients in the COSMIC database, however, this affected codon has been reported in cases of pancreatic cancer (Catalogue of Somatic Mutations in Cancer). In addition, we found a mutation on exon 14, which is similar to previous studies (Papaemmanuil et al., 2011; Cui et al., 2012; Patnaik et al., 2012; Seo et al., 2014; Donaires et al., 2016). Codon E622D was found in 1 patient leading to a change in amino acid from glutamic acid to aspartic acid, and another mutation found in another patient was codon R625C, resulting in a change from arginine to cysteine. The final mutation found on exon 14 was codon H662Q, leading to a change in amino acid from histidine to glutamine. Mutational hotspots of the *SF3B1* gene were found clustered in the fourth, fifth and sixth of the C-terminal HEAT domains (Papaemmanuil et al., 2011; Darman et al., 2015). In missense mutations, the amino acid substitutions may change the size and/or polarity of the side chains of protein structure and can lead to disease. Papaemmanuil (2011) reported that the mutation in the *SF3B1* gene is involved in the pathogenesis of MDS (Papaemmanuil et al., 2011).

To our knowledge, there is no report about the *SRSF2 *gene mutation in the Thai population. We found the frequency of mutation in upper northern Thai patients was 7.3%. This frequency is lower than those in some previous reports. In western populations frequencies differ, for example *SRSF2*^mut^ were found in 12.4% (24/193) of German MDS patients (Thol et al., 2012). In an Asian population, Wu et al., (2012) found the mutation in 14.6% (34/233) of Taiwanese MDS patients (Wu et al., 2012). In contrast, the frequency found by the current study was higher than those found among Chinese MDS patients (4.6% (5/108)) (Lin et al., 2014b). Our study detected the following 2 patterns of missense mutations in 4 patients: P95H (3/4) and P95L (1/4). P95 was the most frequent codon mutation which is consistent with the results of previous studies (Thol et al., 2012; Wu et al., 2012; Kang et al., 2015). The P95H and P95L mutations resulted in proline to histidine and leucine substitutions, respectively. Since SRSF2 protein needs P95 in order to bind to the target RNA, a mutation of P95 affected the function of this protein which may result in the reduction of the RNA binding affinity of the SRSF2 protein (Wu et al., 2012). Previous studies have demonstrated that not only missense mutations but also frameshift deletions have been found in MDS patients. Wu et al., (2012) found that 26.5% (9/34) of *SRSF2*^mut^ in Taiwanese MDS patients were deletion mutations (Wu et al., 2012). Our study found only missense mutations in the Thai population, which may be due to the genetic background and also the limit in number of the test population (n = 55). Further studies with a larger sample size might provide more evidence of the pattern of mutation in the *SRSF2* gene in MDS patients. 

This study additionally analyzed the correlation between the clinical data and *SF3B1*^mut^ and *SRSF2*^mut^ in MDS patients. Similar to prior studies, patients who carried the *SF3B1*^mut^ were found to have significantly lower hemoglobin level (*p* = 0.023) and a higher platelet counts (*p* = 0.047) (Cui et al., 2012; Damm et al., 2012; Rujirachaivej et al., 2018). As was found in some studies which demonstrated that *SF3B1*^mut^ showed a direct correlation with RS (Malcovati et al., 2011; Papaemmanuil et al., 2011; Damm et al., 2012), this study also found that *SF3B1*^mut^ mostly occurred in patients with RS, however, statistically it was a borderline significant difference (*p* = 0.050). Several previous studies demonstrated that patients with* SF3B1*^mut^ had a favorable prognosis (Malcovati et al., 2011; Papaemmanuil et al., 2011; Cui et al., 2012), but there was a study which reported that *SF3B1*^mut^ had no relevance as regards a favorable prognosis (Damm et al., 2012). The conflicting results possibly indicate the heterogeneity of the disease, the selection of analytical variables or co-occurrence with mutations in other genes. There are many current studies concerning the mutational co-occurrence of splicing machinery genes and genes involved in epigenetic regulation of transcription (*ASXL1*, *EZH2*, and *DNMT3A*) (Damm et al., 2012; Thol et al., 2012; Martin et al., 2017). There was a statistically significant difference in shorter overall survival and higher risk of AML progression in patients with co-occurrence of *SF3B1* and *DNMT3A* mutation than those with *SF3B1*^mut^ but *DNMT3A* wild type (Martin et al., 2017). An investigation focusing on Thai MDS patients needs to be carried out regarding this correlation in further studies.

The correlation of the clinical impact with *SRSF2*^mut ^was also evaluated in this study. Wu et al., (2012) demonstrated that patients with *SRSF2*^mut^ showed a strong association with male sex (Wu et al., 2012). However, this correlation was not observed in our study, although, 75% of the *SRSF2*^mut^ patients were male. In addition, the patients with *SRSF2*^mut^ tended to have a higher percentage of blasts than those without mutation (*p* = 0.074). Two of the patients with *SRSF2*^mut^ were found in the MDS-EB-2 subtype and one in the transformation to AML group. The high percentage of BM blasts is an unfavorable factor, giving patients an increased probability of progression to AML. Previous studies indicated that *SRSF2*^mut^ had a strong unfavorable impact in MDS patients (Thol et al., 2012; Wu et al., 2012; Lin et al., 2014b). The *SRSF2 *gene encodes for the serine/arginine rich splicing factor 2 (SRSF2), which involves the splicing E/A complex in the early stage of spliceosome assembly and is involved in the regulation of genomic stability (Xiao et al., 2007). Therefore, a mutation in the *SRSF2* gene might contribute to an adverse prognosis in MDS (Thol et al., 2012). 

In summary, this study showed that the frequencies of *SF3B1*^mut^ and *SRSF2*^mut ^were different from other populations previously studies. We found a co-mutation of Q699H and K700E which has not been previously reported as occurring in MDS patients in the COSMIC database. Patients with the *SF3B1*^mut^ were associated with lower hemoglobin levels, and a higher platelet count. *SRSF2*^mut^ patients appear to have a higher percentage of bone marrow blasts which increases the probability of progression to AML. The information pertinent to *SF3B1*^mut^ and *SRSF2*^mut^ and their possible correlation with clinical features in MDS in an upper northern Thai population might be of further use in diagnosis and prognosis in this patient group. Further studies with a larger sample size might provide more information of gene mutation in MDS patients.

## Conflict of Interest

The authors declare no conflicts of interest.”
